# Molecular Characterization of the Recently Emerged Poultry Pathogen *Ornithobacterium rhinotracheale* by Multilocus Sequence Typing

**DOI:** 10.1371/journal.pone.0148158

**Published:** 2016-02-01

**Authors:** Susann Thieme, Kristin Mühldorfer, Dörte Lüschow, Hafez M. Hafez

**Affiliations:** Institute of Poultry Diseases, Freie Universität Berlin, Berlin, Germany; The University of Hong Kong, HONG KONG

## Abstract

*Ornithobacterium rhinotracheale* (ORT) is an economically important bacterial pathogen of turkeys and chickens worldwide. Since its first detection, a variety of typing methods have been used to gain basic knowledge about the bacterial population structure, an issue that still needs to be addressed. Serological characterization revealed at least 18 different serotypes (A-R) with ORT of serotype A to be predominate among poultry. This study aimed to establish a multilocus sequence typing (MLST) scheme for ORT that could easily be used by other laboratories and allows for worldwide comparison of sequence data. For this purpose, 87 ORT strains from different poultry hosts, geographical origins, years of isolation and serotypes were included in the analysis to identify correlations. Fourteen different sequence types (ST) were found. The most common ST1 was identified in 40 ORT strains from turkeys and chickens on 4 continents and in 3 different European countries. Together with ST9, both STs represented over three quarters (77%) of ORT strains used in the MLST analysis and included strains of frequently cross-reacting ORT serotypes A, E and I. Nine STs were only represented by one ORT strain and might indicate possible avian host, disease or serotype-specific relationships. In contrast, discrepancies between serotype and phylogenetic relatedness were clearly demonstrated by ORT strains that belonged to identical serotypes but differed in their ST. The overall identified low genetic diversity among strains isolated from turkeys and chickens independent of host and geographical origins suggests that ORT has only recently been introduced into domestic poultry and dispersed worldwide.

## Introduction

*Ornithobacterium rhinotracheale* (ORT) has become an important pathogen of turkeys and chickens since its first detection and classification in the early 1990s [1, 2]. Clinical symptoms of infected birds range from respiratory disease, arthritis and increased mortality to growth retardation and decreased egg production. Main pathological findings are fibrinopurulent airsacculitis and pneumonia, sometimes paired with pericarditis and perihepatitis [3]. The severity of ORT infections increases strongly by predisposing factors, such as poor management, high stocking density and inadequate ventilation [3] as well as coinfections with other respiratory pathogens [4–6].

ORT is an emerging bacterial pathogen of global concern in poultry production and has already been isolated in the USA, Brazil, France, the Netherlands, Germany, South Africa, Israel, Japan, Taiwan and even more countries [7–10]. A wide variety of birds including economically important poultry species as well as wild birds like pigeons, pheasants, partridges, falcons and rooks [1, 7, 11, 12] are susceptible to ORT infection or have been found to carry ORT.

Different typing methods have been attempted for characterization of ORT strains and epidemiological investigations. Serological typing of ORT strains by agar gel precipitation test (AGP) is a very practical and common method that was established in 1997 for immunological purposes [9]. Since then 18 different serotypes (A-R) have been reported [13, 14], with A being the most predominate serotype of ORT in poultry. About 95% of ORT isolated from chickens and more than 50% of ORT strains from turkeys belong to serotype A [9, 15]. Cross-reactions between 2 or more ORT serotypes such as A, E and I are common and some strains are not typeable with available antisera [15, 16].

Amonsin et al. [17] were first to investigate the molecular epidemiology of 55 ORT strains from avian hosts in 8 countries on 4 continents by comparison of 3 different methods. The results of multilocus enzyme electrophoresis (MLEE), repetitive sequence based polymerase chain reaction (rep-PCR) and 16S ribosomal RNA (rRNA) gene analyses indicated that ORT strains were genetically closely related independent of hosts and geographic origins. This observation was confirmed by ribotyping results of Leroy-Setrin et al. [18], who characterized 23 ORT strains from 5 different poultry species in France also by plasmid and by random amplified polymorphic DNA (RAPD) analyses. Only RAPD analysis provided good level of discriminatory power indicating 3 different clusters of ORT strains without obvious host-specific differences. Due to the lack of serotype information, a possible relationship was not investigated [18]. A subsequent RAPD analysis of 6 field strains from chickens in Turkey [19] included strains of serotypes A to E (this study RefA-RefE), but did not find clear genotype-serotype correlations. Similar results were obtained for 117 field strains of different serotypes from France and Germany by comparing their RAPD profiles with those of different serotype reference strains (this study RefA-RefQ) [20]. The author concluded that only strains of serotype A might produce a reliable fingerprinting by RAPD-PCR. At least, the comparison of 16S rRNA and OR01 gene analyses [15] and pulsed-field gel electrophoresis (PFGE) [21] results of several ORT field strains with those of ORT serotype reference strains (this study RefA-RefQ) showed consistent genotype-serotype relationships.

As indicated by the results of previous studies, different methods have been used for molecular characterization of ORT. Many included only small numbers of ORT strains, strains representing one poultry host or geographical origin or strains without serotype information, making comparisons between laboratories difficult. Consequently, relevant questions on the population genetic structure and epidemiology of ORT infections still need to be addressed, as for example, to understand the molecular basis of serotype cross-reactivity and their relevance for disease prevention [22, 23].

MLST has been frequently used as powerful tool for reliable molecular characterization of bacteria and fungi, and particularly to address epidemiological questions [24]. The analysis is highly standardized by targeting a set of different gene loci that are distributed on the whole genome of the bacterium. MLST results are unambiguous, highly reproducible and portable, and easily allow the comparison of sequence data together with background information from bacterial strains on a broader context and between laboratories worldwide [25].

The present study aimed to establish MLST as an advanced alternative typing method for ORT. Relationships of possible importance in the epidemiology of ORT infections were investigated by molecular characterization of a variety of strains of different serotypes that have been collected between 1983 and 2014 from different avian hosts and geographical origins.

## Materials and Methods

### Bacterial strains

**Fig 1:**
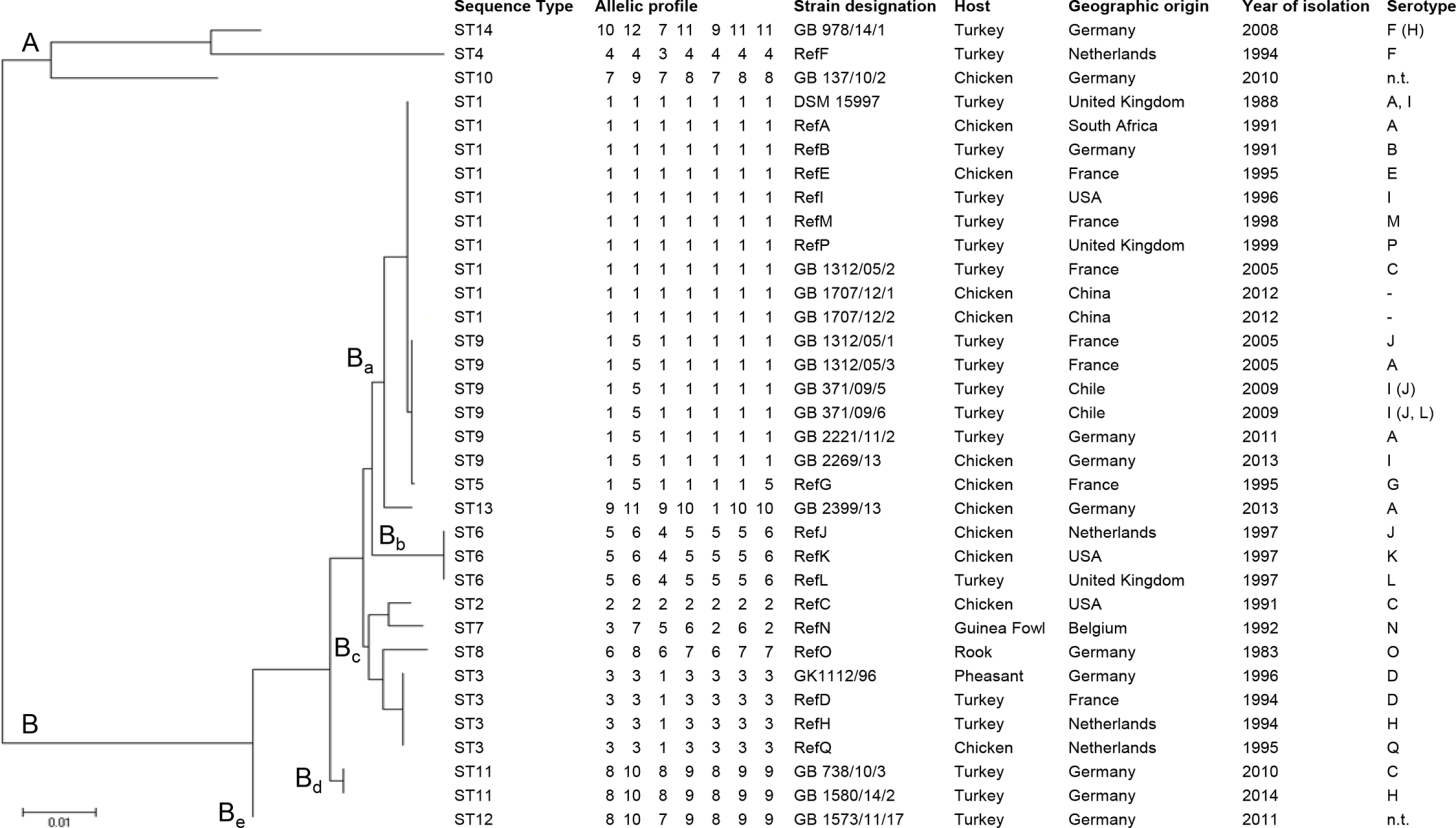
Phylogenetic tree showing the relatedness of 34 representative ORT strains generated from MLST sequences by using the maximum likelihood method of MEGA6 [26]. ORT strains included in the phylogenetic analysis comprised 17 reference strains of serotypes A-Q (RefA-RefQ), 16 field strains mainly from turkeys and chickens of different geographic origins and the ORT type strain DSM 15997. Two main clusters (A and B) and 5 subclusters (Ba-Be) were indicated. Details on sequence type (ST), allelic profile, strain identification (ID), host, geographic origin and serotype were provided. ORT strains that could not be typed with available antisera A to L were indicated by 'n.t.'. Slight serotype cross-reactions in the agar gel precipitation test are given in parentheses. For ORT field strains from China (GB 1707/12), only DNA was available for MLST analysis and the serotype has not yet been determined.

Eighty-seven strains of previously identified ORT were used for the development of MLST comprising 17 ORT reference strains of serotypes A-Q (RefA-RefQ), 69 field strains from turkeys and chickens of different serotypes, geographical origins and years of isolation as well as the ORT type strain DSM 15997 (complete genome available under accession number NC_018016). The ORT field strains have previously been serotyped using agar gel precipitation test as formerly described [14, 16]. Details of all 87 ORT strains are provided as S1 Table. As several ORT strains were identical in their partial gene sequences, 34 out of 87 ORT strains were selected as representatives for presentation of the MLST outcome (Table 1) and phylogenetic analysis (Fig 1). The 34 ORT strains included the ORT type strain DSM 15997, 17 ORT serotype reference strains (RefA-RefQ) and 16 ORT field strains to enable the comparison of different characteristics (serotype, host, geographical origin and year of isolation) possibly associated with MLST results.

**Table 1:** Details of 34 representative strains used for multilocus sequence typing of *Ornithobacterium rhinotracheale*.

ORT strain	Strain designation	Year of isolation	Host	Geographic origin	Serotype
**Type strain**	DSM 15997	1988	Turkey	United Kingdom	A, I
**RefA**	B3263/91	1991	Chicken	South Africa	A
**RefB**	GGD1261/91	1991	Turkey	Germany	B
**RefC**	ORV K91-201	1991	Chicken	USA	C
**RefD**	ORV 94084 nr.2	1994	Turkey	France	D
**RefE**	O-95029 nr.12229	1995	Chicken	France	E
**RefF**	ORV 94084 K858	1994	Turkey	The Netherlands	F
**RefG**	O-95029 nr.16279	1995	Chicken	France	G
**RefH**	E-94063 4.2.	1994	Turkey	The Netherlands	H
**RefI**	BAC-96-8334	1996	Turkey	USA	I
**RefJ**	O-97091 HEN81-2	1997	Chicken	The Netherlands	J
**RefK**	BAC970321101 5m	1997	Chicken	USA	K
**RefL**	O-97071 BUT 2237	1997	Turkey	United Kingdom	L
**RefM**	TOP 98036 98.4500	1998	Turkey	France	M
**RefN**	TOP 99023 LMG13114	1992	Guinea Fowl	Belgium	N
**RefO**	TOP 99023 LMG11553	1983	Rook	Germany	O
**RefP**	TOP 99090 may 71	1999	Turkey	United Kingdom	P
**RefQ**	O-95256 sp 1507	1995	Chicken	The Netherlands	Q
**Field strains**	GK 1112/96	1996	Pheasant	Germany	D
	GB 1312/05/1	2005	Turkey	France	J
	GB 1312/05/2	2005	Turkey	France	C
	GB 1312/05/3	2005	Turkey	France	A
	GB 978/14/1	2008	Turkey	Germany	F (H)^a^
	GB 371/09/5	2009	Turkey	Chile	I (J) ^a^
	GB 371/09/6	2009	Turkey	Chile	I (J, L) ^a^
	GB 137/10/2	2010	Chicken	Germany	n. t.
	GB 738/10/3	2010	Turkey	Germany	C
	GB 1573/11/17	2011	Turkey	Germany	n.t.
	GB 2221/11/2	2011	Turkey	Germany	A
	GB 1707/12/1	2012	Chicken	China	^b^
	GB 1707/12/2	2012	Chicken	China	^b^
	GB 2269/13	2013	Chicken	Germany	I
	GB 2399/13	2013	Chicken	Germany	A
	GB 1580/14/2	2014	Turkey	Germany	H

RefA-RefQ: ORT strains of serotypes A to Q that have been used for production of reference antisera for serological typing of ORT field strains [9]. n.t.: ORT strain that could not be typed with available antisera A to L.

^a^ Slight serotype cross-reactions of ORT strains are given in parentheses.

^b^ The serotype of the respective ORT strain was not determined, as only DNA was available for MLST analysis.

### DNA extraction

All ORT strains were cultured from frozen bacterial stocks onto Columbia agar plates with 7% sheep blood and were incubated at 37°C under a 5% CO_2_ atmosphere for 24–48 hours. DNA extraction was performed by heat cell lysis for Gram-negative bacteria. A loopful of bacterial colonies dissolved in 200 μl of nuclease free water was heated at 98°C for 10 minutes followed by 10 minutes of centrifugation (20,000 × g). The supernatant was used as DNA template for PCR analysis and stored at -20°C until further use.

### Selection of housekeeping genes for MLST

A set of 7 different housekeeping genes were selected for MLST of ORT (Table 2) based on previous studies of MLEE analysis of ORT [17] and MLST analysis of *Pasteurella multocida* [27] because of high similarities between both bacterial species [2]. Three genes (*adk*, *mdh*, *pgi*) were present in both studies, whereas genes *aroE* and *fumC* were selected from MLEE analysis and genes *gdhA* and *pmi* from MLST of *P*. *multocida* (Table 2). Gene loci are scattered across the main chromosome to ensure independence from each other.

**Table 2:** Housekeeping genes, primers and corresponding gene fragments used for multilocus sequence typing of *Ornithobacterium rhinotracheale*.

Gene	Protein product	Primer (5’→3’)^a^	Fragment size used for MLST	MLEE of ORT [17]	MLST of *P*. *multocida* [27]
*adk*	Adenylate kinase	F: GGCAGTGGAAAAGGAACTCA R: TCTAAACTTCCTTCGCCGTTT	393 bp	X	X
*aroE*	Shikimate 5-dehydrogenase	F: GGACTCATCGGCAGAAACAT R: TGATGTTGGCATCTTGTGCT	489 bp	X (SKD)^b^	
*fumC*	Fumarase, class II	F: CACGCCACAAGGTTATGATG R: TAAACGCACGGCTTCTTCTT	489 bp	X (FU2) ^b^	
*gdhA*	Glutamate dehydrogenase/ Leucine dehydrogenase	F: TCTGGTAGAGCACCAAACCA R: GCTTGTTTTGCAACCACTCA	480 bp		X
*mdh*	Malate dehydrogenase (NAD)	F: CGCGAAGAATTAATCGGAAC R: CTCTTACTTGCGCAACAGCA	519 bp	X	X
*pgi*	Glucose-6-phosphate isomerase	F: AAAGCGACATTGCCAAACAT R: TTTCGAGTTCCGCTCTCACT	492 bp	X	X
*pmi*	Phosphomannose isomerase	F: TGATGTGCAAGGCAATGTTT R: CTGTGTCGAGCGAAATGCTA	489 bp		X

^a^ F–forward primer; R–reverse primer.

^b^ Abbreviations used by Amonsin et al. [17] for the respective enzyme.

### Gene amplification and sequencing

PCR analysis and partial sequencing of 7 housekeeping genes was performed on 86 out of 87 ORT strains. Gene sequences from the ORT type strain DSM 15997 were taken directly from GenBank (http://www.ncbi.nlm.nih.gov/genbank/index.html).

The primer sets used for MLST analysis (Table 2) were created based on the genomic sequence of ORT type strain DSM 15997 using the primer designing tool Primer 3 [28]. For the ORT reference strain of serotype F (RefF), a different reverse primer (5’-TCRTTCCATTTRTTTTGTCCTT-3’) was used for PCR amplification and partial sequencing of the housekeeping gene *adk* to yield the desired amplicon.

PCR was carried out using Ready-To-Go PCR beads (GE Healthcare, Freiburg, Germany) with 25 μl reaction volumes containing 1 μl of bacterial DNA and 1 μl of the respective forward and reverse primers (25 pmol/μl). PCR cycler conditions for all genes were the following: initial denaturation at 94°C for 5 minutes followed by 30 cycles of denaturation at 94°C for 30 seconds, annealing at 52°C for 60 seconds and extension at 72°C for 90 seconds. The final extension at 72°C for 7 minutes completed the PCR. Clean-up of PCR products and Sanger sequencing in both directions were performed by microtitre plate sequencing at LGC Genomics, Berlin, Germany.

### MLST analysis

Raw sequence chromatograms were visually screened for quality with the Chromas Lite software (version 2.01; Technelysium Pty Ltd, South Brisbane, Australia) and sequences were uploaded to BioNumerics (version 7.1.; Applied Maths, Sint-Martens-Latem, Belgium).

For each gene locus, nucleotide sequence differences between strains were identified by alignment and distinct alleles were numbered in ascending order. The composition of 7 allele numbers formed the allelic profile of the ORT strain and the subsequent ST in order of identification (ST1, ST2,…). Thus, each unique allelic profile represented a new ST.

Discriminatory power was calculated with the formula of Simpson’s index of the discriminatory ability (D) as previously described [29] by using the tool http://insilico.ehu.es/mini_tools/discriminatory_power/index.php. The discriminatory ability describes the capability of a typing method to identify strains to be different from each other. The sum ranges from 0.0 to 1.0 and estimates the probability to obtain different types for two strains randomly selected from a sample. The higher the index the more discriminatory the method.

The index of association (*I*_*A*_) describes the level of genetic recombination and was calculated as described by Maynard Smith et al. [30] using the START2 software [31]. A value of zero reflects no association between gene loci (linkage equilibrium). If the index is significantly different from zero more recombination takes place in the population.

For each of the 34 representative ORT strains, partial sequences of the 7 alleles were concatenated and a phylogenetic tree was built with the MEGA software (version 6) using the maximum likelihood method [26].

### Nucleotide sequence accession numbers

The DNA sequences of the distinct alleles at the 7 loci of ORT strains used in this study have been deposited in GenBank under accession nos. KP775847-KP775856 (*adk*), KP775857-KP775868 (*aroE*), KP775869-KP775877 (*fumC*), KP775878-KP775888 (*gdhA*), KP775889-KP775897 (*mdh*), KP775898-KP775908 (*pgi*) and KP775909-KP775919 (*pmi*).

Information about primer sequences, PCR conditions, allele sequences, sequence types and isolates have been made available at the ORT MLST website (http://pubmlst.org/orhinotracheale/) sited at the University of Oxford [32].

## Results and Discussion

The purpose of this study was to enable the characterization of ORT strains under standardized conditions by developing a MLST scheme, which could easily be used and expanded by other laboratories, and finally allows for worldwide comparison of sequence data. To approach this idea, 7 housekeeping genes were identified from previous studies on ORT [17] and *P*. *multocida* [27], and newly developed primer pairs were used for partial sequencing of ORT strains representing different poultry hosts, geographical origins, years of isolation and serotypes.

### MLST analysis

The length of the gene sequences used for MLST varied from 393 bp (*adk* gene) to 519 bp (*mdh* gene). No deletions or insertions were identified in any of the sequences. For 87 ORT strains, 9 to 12 alleles for each gene locus were distinguished with a mean number of 10.43.

The number of polymorphic sites varied notably between the 7 housekeeping genes. For example, a maximum number of 22 and 105 polymorphic sites were identified within the partial sequences of genes *pmi* and *gdhA*, respectively (Table 3). Most nucleotide polymorphisms were seen in 3 strains, namely RefF (turkey, the Netherlands), GB 978/14/1 (turkey, Germany) and GB 137/10/2 (chicken, Germany). The number of polymorphic sites among *gdhA* gene sequences decreased markedly from 105 to 10 if these 3 strains were excluded from the analysis (Table 3).

**Table 3:** Maximum number of alleles and polymorphic sites per gene locus.

Gene	Number of different alleles	Number of polymorphic sites	Number of polymorphic sites without cluster A
*adk*	10	51	14
*aroE*	12	73	25
*fumC*	9	58	24
*gdhA*	11	105	10
*mdh*	9	73	18
*pgi*	11	59	12
*pmi*	11	22	12
**Total**	**14 different STs**	**441**	**115**

Fourteen different STs were identified among the 87 ORT strains by MLST, which is low compared to results of other bacterial species [27, 33–35]. Nine sequence types were only represented by one ORT strain (Fig 1). The most common ST1 was identified in 40 (46%) ORT strains and included 6 different serotype reference strains (RefA, RefB, RefE, RefI, RefM, RefP) as well as type strain DSM 15997. Together with the closely related ST9, which differed only in one allele from ST1, both STs represented 77% of ORT strains. Sequence types 3, 6 and 11 included 4, 3 and 4 strains, respectively. The value of the discriminatory power was 0.6937, which is low compared to MLST indices of other bacterial species that offered discriminatory powers close to 1.0 [27, 35, 36].

The index of association (*I*_*A*_) calculated for all 87 strains analyzed was 4.86. Significant linkage disequilibrium was detected indicating clonal population structures. Limited genetic heterogeneity of ORT has been suggested by Amonsin et al. [17] who characterized different ORT strains from poultry hosts of various geographical origins by MLEE, rep-PCR and 16S rRNA gene analyses. The authors supposed that all strains from domesticated poultry belong to a single clonal complex. Similar results have also been shown by whole-cell protein analyses. Hung and Alvarado [37] analyzed 25 ORT strains from outbreaks of respiratory disease in chickens from Peru and found identical SDS-PAGE protein patterns.

Two distinct phylogenetic clusters A and B were identified in the phylogenetic tree generated from MLST sequences (Fig 1). Three ORT strains (RefF, GB 978/14/1 and GB 137/10/2) from turkeys and chicken in Germany and the Netherlands formed cluster A and differed considerably from the vast majority of ORT strains (all cluster B) included in this study. Interestingly, they represented 2 strains of the rarely identified serotype F and one field strain that was not typeable with available antisera. Thus, cluster B included 84 (96.6%) of the 87 investigated ORT strains and was divided into 5 closely related subclusters (Ba-Be).

### Relationships between STs and hosts

Eighty-four out of 87 ORT strains (96.6%) were isolated from turkeys or chickens (S1 Table) and did not reveal clear species-specific correlations based on MLST results. Sequence types, which were identified more than once (ST1, ST3, ST6 and ST9), comprised strains from both turkey and chicken. The only exception was ST11 that solely included field strains from turkeys.

As mentioned before, high genetic similarity among ORT strains independent of poultry hosts has been reported by Amonsin et al. [17]. Van Empel et al. [38] showed that ORT strains isolated either from turkeys or chickens produce similar disease symptoms in cross-infection experiments. However, rep-PCR results of Amonsin et al. [17] indicated that ORT clones infecting passeriform birds were genetically distinct from clones infecting galliform birds. Clear host-specific differences between ORT strains from non-galliform birds such as pigeons and those from poultry species have also been shown by 16S rRNA analysis [7].

In this study, 3 ORT strains originated from pheasant, guinea fowl and rook. Both ORT strains from guinea fowl (RefN) and rook (RefO) formed unique STs (ST7 and ST8), while the pheasant-derived field strain (serotype D) belonged to ST3 together with reference strains of serotypes D, H and Q isolated from turkey or chicken. The 3 strains isolated from non-commercial-poultry host species or respective from a passeriform bird were included in subcluster Bc of the main cluster B (Fig 1).

### Relationship between STs and geographic origin

The MLST analysis included strains from 9 different countries and 4 continents (S1 Table) that were widely distributed across STs and phylogenetic clusters (Fig 1). ORT strains from different geographical origins were assigned to identical STs, namely ST1, ST3, ST6 and ST9, indicating that a specific ST is not linked to geographical origin. For example, subcluster Ba included 68 (78.2%) ORT strains of 4 STs from 7 countries on 4 continents. Such a global distribution of few STs that grouped phylogenetically very close together provides further evidence of highly clonal bacterial population structures. Likewise, analyses of either outer membrane or total proteins demonstrated high similarities between polypeptide profiles of ORT strains originating from various countries worldwide [1, 13].

Interestingly, ST11 was identified in 4 field strains that have been isolated in different years from turkey flocks in Germany (S1 Table). This relationship to bacterial origin might be biased because of the randomized dataset used for the development of MLST. However, PFGE results of Popp [21] indicated a possible relation between geographical origin and genetic homogeneity of ORT strains. Her analysis included 11 field strains from Hungary that together with only one strain from Germany had identical PFGE patterns and represented a single clonal complex of only 41% similarity to other ORT strains.

### Relationship between STs and year of isolation

The 87 ORT strains of this study were collected between 1983 and 2014. Four strains that have been isolated between 1994 and 1996, and 3 strains from 1997 were assigned to ST3 and ST6, respectively. Strains of ST3 clustered together with 3 strains of ST2, ST7 and ST8 (one strain each). All 7 ORT strains formed subcluster Bc and have been collected in a short period of time between 1991–1996 or rather in 1983 (ST8). In contrast, ORT strains of ST1 (n = 40) and ST9 (n = 27) ranged more widely in their years of isolation from 1988 to 2013 and 2005 to 2013, respectively (S1 Table). To date, there are no earlier studies that investigated a possible epidemiological relationship between ORT strains of different years.

### Relationship between STs and serotypes

The ORT reference strains of serotypes A-Q (RefA-RefQ) were included in the MLST analysis as well as different field strains that have been characterized previously by serotyping. The predominant ORT serotypes A, B, E or I were identified in 58 (67%) strains investigated in this study. It has been shown that these 4 serotypes frequently cross-react in AGP tests ([15], Hafez et al., unpublished data). Based on MLST results, they belonged to 3 different STs (ST1, ST9 and ST13) and formed subcluster Ba (Fig 1). The serotype strain RefG alone formed ST5 but differed only in 1 or 2 alleles from strains of ST9 or ST1 and belonged to subcluster Ba. Both STs 9 and 1 included the majority of ORT field strains that slightly cross-reacted with 3 to 5 different serotypes including serotype G (S1 Table). In general, STs which were represented by 2 or more ORT strains such as ST1, ST3, ST6, ST9 and ST11, included more than one ORT serotype. Two ORT strains of serotype F from turkeys were investigated by MLST that clearly differed in their STs, but belonged both to cluster A. Strains RefN and RefO were the only representatives for ORT serotypes N and O and formed unique STs (ST7 and ST8, respectively) within the major cluster B.

Serotype-specific relationships have been indicated by previous studies using PFGE [21], 16S rRNA and Or01 [15] gene analyses or RAPD-PCR [19, 20] for characterization of ORT. In the PFGE analysis serotype strains A-Q were identical with RefA-RefQ used in the present study. Consistent with MLST results, strains of serotypes A, E, I and P (this study ST1) as well as strains RefD, RefH and RefQ (this study ST3) grouped together based on their PFGE patterns [21]. Analyses of partial 16S rRNA and Or01 gene sequences by Numee et al. [15] also included ORT serotype strains A-H (this study RefA-RefH) and were in agreement with both MLST and PFGE results. Again, strains RefA, RefB and RefE clustered together in the neighbor-joining tree, and strains RefD and RefH showed close relationship to each other. Likewise, Ozbey et al. [19] found that some strains of serotype A shared their RAPD profile with those of serotype B and E. Only the serotype reference strain C (this study RefC) had a unique RAPD profile. The relationship between strains of serotypes A and B as well as the distinct RAPD profile of serotype C were confirmed by Waldow [20].

This study included 3 ORT strains of serotype C that were assigned to distinct STs and subclusters Ba, Bc and Bd based on nucleotide differences in all 7 gene loci (Fig 1). RefC formed its own ST (ST2), whereas both field strains shared identical STs with other strains (ST1 and ST11). Consistent results were found for 2 strains of serotype J that belong to different STs and subclusters (Fig 1). As the molecular basis for serological variations of ORT remains unknown, the most likely explanation might be the transfer of genes between strains encoding serotype specificity. Horizontal spread of capsular genes leading to identical serotypes in genetically diverse bacterial strains has been discussed for other bacteria like *Streptococcus* species [39–41]. Based on MLST results and serotype information, it is most likely that serotype exchange frequently occurs among ORT strains from turkeys. Only 2 STs were identified by the MLST scheme that represented the majority of ORT and included most common serotypes as well as frequently cross-reacting strains.

## Conclusion

The MLST scheme presented here can provide an unambiguous, reproducible and portable typing system to stepwise gain more insights into the population structure of ORT by identifying the extent of recombination. It has limited discriminatory power, which is most likely associated with the observed low genetic heterogeneity and clonal population structure of ORT that originated from poultry species. In general, ORT strains from turkeys or chickens in different geographical regions were not separated by MLST and assigned to identical STs. However, 3 strains differed markedly from the vast majority suggesting that ongoing analyses of more strains could increase the discriminating ability of this method. The inclusion of ORT strains from non-poultry host species will enhance our understanding whether these birds may act as vectors in disease transmission. On a future perspective, sequence data from laboratories worldwide together with background information on specific disease symptoms or virulence can be very helpful to address epidemiological questions and may lead to improvements for the control of ORT infections.

## Supporting Information

S1 Table Details of the 87 strains used for multilocus sequence typing of *Ornithobacterium rhinotracheale*. RefA-RefQ: ORT strains of serotypes A to Q that have been used for production of reference antisera for serological typing of ORT field strains [9]. Slight serotype cross-reactions of ORT strains are given in parentheses. n.t.: ORT strain that could not be typed with available antisera A to L. ^a^ The serotype of the respective ORT strain was not determined, as only DNA was available for MLST analysis.
(PDF)
