# Correction: Molecular Characterization of the Recently Emerged Poultry Pathogen *Ornithobacterium rhinotracheale* by Multilocus Sequence Typing

**DOI:** 10.1371/journal.pone.0163401

**Published:** 2016-09-15

**Authors:** Susann Thieme, Kristin Mühldorfer, Wael Gad, Dörte Lüschow, Hafez M. Hafez

Dr. Wael Gad should be listed in the author byline instead of the Acknowledgments. He should be listed as the third author, and his affiliation is 1: Institute of Poultry Diseases, Freie Universität Berlin, Berlin, Germany. The contributions of this author are as follows: Conceptualization, Methodology, Investigation.

The correct citation is: Thieme S, Mühldorfer K, Gad W, Lüschow D, Hafez HM (2016) Molecular Characterization of the Recently Emerged Poultry Pathogen *Ornithobacterium rhinotracheale* by Multilocus Sequence Typing. PLoS ONE 11(2): e0148158. doi:10.1371/journal.pone.0148158
