# Supplementary material for: Molecular Characterization of the Recently Emerged Poultry Pathogen Ornithobacterium rhinotracheale by Multilocus Sequence Typing
Source: PLoS One. 2016 Feb 1;11(2):e0148158. doi: 10.1371/journal.pone.0148158 (PMC4734695; doi:10.1371/journal.pone.0148158)
Supplement: S1 Table — RefA-RefQ: ORT strains of serotypes A to Q that have been used for production of reference antisera for serological typing of ORT field strains [9]. Slight serotype cross-reactions of ORT strains are given in parentheses. n.t.: ORT strain that could not be typed with available antisera A to L. a The serotype of the respective ORT strain was not determined, as only DNA was available for MLST analysis. (PDF) [file pone.0148158.s001.pdf]

Supplemental table

No.	Strain designation	Year of isolation	Host	Geographic origin	Serotype	Sequence Type
			**REFERENCE STRAINS**			
1	DSM 15997	1988	Turkey	United Kingdom	A, I	1
2	RefA	1991	Chicken	South Africa	A	1
3	RefB	1991	Turkey	Germany	B	1
4	RefC	1991	Chicken	California, USA	C	2
5	RefD	1994	Turkey	France	D	3
6	RefE	1995	Chicken	France	E	1
7	RefF	1994	Turkey	The Netherlands	F	4
8	RefG	1995	Chicken	France	G	5
9	RefH	1994	Turkey	The Netherlands	H	3
10	RefI	1996	Turkey	Minnesota, USA	I	1
11	RefJ	1997	Chicken	The Netherlands	J	6
12	RefK	1997	Chicken	USA	K	6
13	RefL	1997	Turkey	United Kingdom	L	6
14	RefM	1998	Turkey	France	M	1
15	RefN	1992	Guinea fowl	Belgium	N	7
16	RefO	1983	Rook	Germany	O	8
17	RefP	1999	Turkey	United Kingdom	P	1
18	RefQ	1995	Chicken	The Netherlands	Q	3
			**FIELD STRAINS**			
19	GK 304/95	1995	Chicken	Germany	A	1
20	GK 1049/95	1995	Chicken	Germany	A	1
21	GK 1112/96	1996	Pheasant	Germany	D	3
22	GB 137/10/1	2010	Laying hen	Germany	A	1
23	GB 137/10/2	2010	Laying hen	Germany	n.t.	10
24	GB 299/10/6	2010	Turkey	Germany	B, E	1
25	GB 299/10/7	2010	Turkey	Germany	E	1

26	GB 299/10/8	2010	Turkey	Germany	n.t.	1
27	GB 404/10/1	2010	Turkey	Germany	n.t.	11
28	GB 404/10/2	2010	Turkey	Germany	I (J)	1
29	GB 596/10/2	2010	Turkey	Germany	A, I	1
30	GB 610/10/1	2010	Turkey	Germany	E	1
31	GB 610/10/2	2010	Turkey	Germany	E	1
32	GB 610/10/3	2010	Turkey	Germany	E	1
33	GB 738/10/1	2010	Turkey	Germany	n.t.	11
34	GB 738/10/2	2010	Turkey	Germany	E	1
35	GB 738/10/3	2010	Turkey	Germany	C	11
36	GB 738/10/4	2010	Turkey	Germany	A	1
37	GB 1032/10/1	2010	Turkey	Germany	B, L	1
38	GB 56/11/1	2011	Turkey	Germany	A	1
39	GB 57/11/1	2011	Turkey	Germany	A	1
40	GB 57/11/2	2011	Turkey	Germany	A	1
41	GB 604/11/3	2011	Turkey	Germany	A, I	9
42	GB 1103/11/1	2011	Turkey	Germany	E (J,K)	9
43	GB 1573/11/16	2011	Turkey	Germany	B (I,J,L)	1
44	GB 1573/11/17	2011	Turkey	Germany	n.t.	12
45	GB 1573/11/18	2011	Turkey	Germany	A (I,J,L)	1
46	GB 2151/11/1	2011	Turkey	Germany	B (I,J)	1
47	GB 2151/11/2	2011	Turkey	Germany	A (B,I)	1
48	GB 2151/11/3	2011	Turkey	Germany	A (B,I)	1
49	GB 2221/11/1	2011	Turkey	Germany	A (K)	9
50	GB 2221/11/2	2011	Turkey	Germany	A	9
51	GB 2221/11/3	2011	Turkey	Germany	A	9
52	GB 365/12/1	2012	Turkey	Germany	B, J	9
53	GB 1538/12/1	2012	Turkey	Germany	A	9
54	GB 2177/13/1	2013	Turkey	Germany	I	9
55	GB 2269/13/1	2013	Chicken	Germany	I	9
56	GB 2399/13/1	2013	Chicken	Germany	A	13
57	GB 2992/13	2013	Turkey	Germany	I	9
58	GB 3031/13/1	2013	Turkey	Germany	E (G, J, K)	1

59	GB 3031/13/2	2013	Turkey	Germany	E (B, G, J, K)	1
60	GB 3074/13	2013	Turkey	Germany	I (B, E, G, K, L)	9
61	GB 3111/13/1	2013	Turkey	Germany	I (B, E, G, J, L)	9
62	GB 3111/13/2	2013	Turkey	Germany	E, G	1
63	GB 3111/13/3	2013	Turkey	Germany	I (B, G)	9
64	GB 978/14/1	2008	Turkey	Germany	F (H)	14
65	GB 1580/14/2	2014	Turkey	Germany	H	11
66	GB 1312/05/1	2005	Turkey	France	J	9
67	GB 1312/05/2	2005	Turkey	France	C	1
68	GB 1312/05/3	2005	Turkey	France	A	9
69	GB 1312/05/8	2005	Turkey	France	I	1
70	GB 1312/05/22	2005	Turkey	France	J	9
71	GB 1312/05/23	2005	Turkey	France	B (C)	1
72	GB 371/09/5	2009	Turkey	Chile	I (J)	9
73	GB 371/09/6	2009	Turkey	Chile	I (J,L)	9
74	GB 371/09/7	2009	Turkey	Chile	I (J,L)	9
75	GB 735/09/10	2009	Turkey	Chile	A	9
76	GB 735/09/12	2009	Turkey	Chile	A	9
77	GB 735/09/13	2009	Turkey	Chile	A	9
78	GB 1707/12/1	2012	Chicken	China	-[a]	1
79	GB 1707/12/2	2012	Chicken	China	-[a]	1
80	GB 1707/12/3	2012	Chicken	China	-[a]	1
81	GB 1707/12/7	2012	Chicken	China	-[a]	1
82	GB 804/13/1	2013	Turkey	United Kingdom	I	9
83	GB 804/13/2	2013	Turkey	United Kingdom	A (I)	9
84	GB 804/13/3	2013	Turkey	United Kingdom	I	9
85	GB 954/13/1	2013	Turkey	United Kingdom	A, I	1
86	GB 954/13/2	2013	Turkey	United Kingdom	A, I	9
87	GB 954/13/3	2013	Turkey	United Kingdom	A, I	9
